# Differential regulatory effects of *Lycium barbarum* polysaccharide components in cyclophosphamide-treated immunosuppressed mice: reshaping of the gut microbiota

**DOI:** 10.1016/j.fochms.2025.100305

**Published:** 2025-10-03

**Authors:** Xin An, Yuan Chen, Yufei Chu, Mengjia Zhang, Ye Liu, Yuejuan Zhao, Shan Wu, Qian Liu

**Affiliations:** a College of Food Science and Technology, Northwest University, 229 Tabai North Road, Xi'an 710069, China; b Inner Mongolia Enterprise Key Laboratory of Dairy Nutrition, Health & Safety, Inner Mongolia Mengniu Dairy (Group) Co. Ltd., Huhhot 011500, China; c Research and Development Center, Xi'an Yinqiao Dairy (Group) Co., Ltd., Xi'an 710075, China; d Shaanxi Functional Food Engineering Center Co.,Ltd, China

**Keywords:** *Lycium barbarum* polysaccharide, Immunomodulatory, Intestinal flora, Short-chain fatty acid, Serum metabolomics

## Abstract

*Lycium barbarum* polysaccharides (LBPs) have recently attracted considerable attention for their immunomodulatory potential. However, their complex structures hinder efficient isolation and purification, making it challenging to identify the active components and establish clear structure-function relationships. We hypothesized that various LBP components exert distinct immunomodulatory effects by modulating gut microbiota composition and host metabolism. In this study, LBP fractions with distinct physicochemical properties were obtained via graded ethanol precipitation and evaluated using a cyclophosphamide (CTX)-induced immunosuppressed mouse model. Immunomodulatory effects and underlying mechanisms were investigated through gut microbiota profiling, short-chain fatty acid (SCFAs) quantification, and serum metabolomics analysis. The results showed that various LBP components alleviated CTX-induced weight loss, protected immune organs, enhanced the secretion of immune-related cytokines, and improved the activity of liver antioxidant enzymes to varying degrees. Various LBP components distinctly reshaped the gut microbiota composition and SCFAs production. Among them, the LBP3 component exhibited the strongest immunomodulatory activity, markedly enhanced acetic and propionic acids concentrations as well as the relative abundance of *Bifidobacterium*, *Lactobacillus*, *Bacteroides*, and *Akkermansia*. Furthermore, Serum metabolomics revealed that LBP3 intervention significantly upregulated metabolite levels, including inosine, pentadecanoic acid, propionylcarnitine, and fucoxanthin. These findings confirm our hypothesis that structurally distinct LBP components exert differential immunomodulatory effects by modulating gut microbiota and host metabolism, thereby providing a theoretical basis for the targeted development of LBP-based functional foods and natural immunomodulators.

## Highlights

• Various LBP components differentially restored immune function.
• LBP3 modulated the gut microbiota and serum metabolomics in immunosuppressed mice.
• LBP3 significantly increased acetic and propionic acids concentrations.

## 1 Introduction

Increasing evidence indicates that immune function is closely linked to the gut microbiota, and modulating the composition of gut microbial communities can indirectly restore impaired immunity (Jia et al., 2025; Park et al., 2025). Therefore, developing safe and effective natural immunomodulators that target the gut microbiota is of great significance for enhancing host immunity and promoting health.

Polysaccharides as bioactive compounds have attracted considerable attention due to their diverse biological activities, including antioxidant, anticancer, hypoglycemic, anti-inflammatory, and immunomodulatory effects (Chen, Gao, et al., 2024; Liu et al., 2024; Yu et al., 2018). Owing to their high molecular-weight (Mw) carbohydrate structures and dietary fiber characteristics, most of plant polysaccharides are resistant to direct degradation or absorption in the stomach and can reach the colon intact, where they serve as fermentation substrates for gut microbes (Ding, Yan, Chen, et al., 2019). Beyond shaping the gut microbial community, polysaccharides can also be degraded into SCFAs by various carbohydrate-active enzymes (CAZymes) secreted by microbes (Zhao et al., 2025). SCFAs not only provide an energy source for the host but also enhance intestinal barrier function, modulate immune responses, and maintain a healthy gut ecosystem by promoting beneficial bacteria and suppressing potential pathogens (Zhu et al., 2024). Consequently, the immunomodulatory effects of polysaccharides largely depend on the “polysaccharide–gut microbiota–host” axis. For instance, previous studies have shown that *Dendrobium officinale* polysaccharides significantly increase the relative abundance of SCFA-producing bacteria (such as *Ruminococcaceae*, *Bacteroides*, and *Lactobacillus*), thereby effectively alleviating DSS-induced colitis (Zhang et al., 2020).

*Lycium barbarum* (goji berry) is both a medicinal and edible plant, rich in polysaccharides, carotenoids, flavonoids, vitamins, and minerals (Berisha et al., 2025; Xiong et al., 2025). These constituents collectively confer antioxidant, antimicrobial, anti-inflammatory, and immunomodulatory activities (Ahmad et al., 2024; Shi et al., 2025). Among them, LBPs—water-soluble polysaccharides accounting for 5–8 % of the fruit's dry weight—are recognized as major bioactive components, exerting antioxidant, barrier-protective, neuroprotective, and gut microbiota-modulating effects (Karen Ruiz-Salinas et al., 2020; Lai et al., 2022; Zhang et al., 2025). Previous studies have shown that LBPs alleviate inflammatory bowel disease by modulating STAT1/STAT6 signaling (Wang et al., 2023), and exert hepatoprotective effects by inhibiting thioredoxin-interacting protein (TXNIP) and suppressing NOD-like receptor 3 (NLRP3) inflammasome activation (Xiao et al., 2014). However, most research has focused on crude polysaccharides (CLBPs), which contain complex and incompletely characterized components. To overcome this limitation, our team employed graded alcohol precipitation to efficiently separate three distinct polysaccharide components from CLBP, with LBP3 being a uniform arabinogalactan, recognized as the primary active ingredient responsible for the anti-aging effects of L. *barbarum* (Gong et al., 2018; Tang et al., 2019). Notably, Cao et al. (2021) showed that LBP3 alleviates DSS-induced chronic colitis by protecting the intestinal barrier and suppressing TLR4-MyD88-NF-ҡB signaling. Our previous study further indicated that various LBP components differentially modulate gut microbiota composition and SCFAs production in normal mice (An et al., 2024). However, their immunomodulatory effects in immunosuppressed mice remain unclear.

Therefore, the present study aimed to determine whether specific LBP components exert distinct immunomodulatory effects by reshaping the gut microbiota and influencing host metabolism. The findings aim to provide deeper insights into the immunomodulatory mechanisms of LBPs and support the targeted development of functional foods or natural immunomodulators featuring arabinogalactan structures.

## 2 Materials and methods

### 2.1 Materials and chemicals

Dried L. *barbarum* fruits were sourced from Jiabao *Lycium barbarum* Trading Co., Ltd. (Ningxia, China). Standards for CTX and SCFAs were obtained from Macklin Biochemical Co., Ltd. (Shanghai, China). All other reagents were of analytical grade.

### 2.2 Isolation and purification of LBPs

Various LBP components were prepared as previously described (Gong et al., 2018). Briefly, CLBP was obtained through hot water extraction, followed by ethanol precipitation, sevag deproteinization, and dialysis. LBP1, LBP2, and LBP3 were isolated by fractionating CLBP with varying concentrations of ethanol (30 %, 50 %, 70 %) [among these, LBP3 exhibited the highest total carbohydrate content (60.2 ± 0.5 %) and the lowest protein content (31.1 ± 1.9 %), and possessed a relatively uniform arabinogalactan structure primarily composed of arabinose (Ara, 44.3 %) and galactose (Gal, 43.8 %), with a Mw of 8.8 × 10^4^ Da] (An et al., 2024).

### 2.3 Animals and experimental design

Sixty male BALB/c mice (23.5 ± 1 g, 7–8-weeks-old) were sourced from Beijing Vital River Laboratory Animal Technology Co., Ltd. (Beijing, China). Mice were housed under controlled conditions (22 ± 2 °C, 55 ± 10 % humidity, 12/12 h light/dark cycle) with free access to clean water and standard chow. All the experimental protocols were authorized by the Animal Ethics Committee of the Laboratory Animal Center of Northwest University (Permit Code: NWU-AWC-20220907 M) and adhered to the National Research Council's Guide for the Care and Use of Laboratory Animals. Every effort was made to minimize animal suffering. After one-week of acclimatization, mice were randomly assigned to six groups (*n* = 10): normal control (NC), model control (CTX), CTX + CLBP, CTX + LBP1, CTX + LBP2, and CTX + LBP3. To ensure comparable baseline conditions, mice were stratified by body weight (±1 g), and within each weight block, they were assigned to one of the six groups using a computer-generated random sequence (Excel RAND () function). Except for the NC group, all mice received intraperitoneal CTX injections (80 mg/kg body weight) daily for three consecutive days to induce immunosuppression. Over the subsequent 24 days, mice in the CTX + CLBP, CTX + LBP1, CTX + LBP2, and CTX + LBP3 groups were gavaged daily with the corresponding polysaccharide components (100 mg/kg body weight), whereas those in the NC and CTX groups received an equal volume of saline. After the final administration, mice were fasted for 12 h with free access to water. All mice were anesthetized, and blood was collected via enucleation of the eyeballs, followed by euthanasia through cervical dislocation. Blood samples were centrifuged (3500 ×*g*, 15 min, 4 °C) to obtain serum. Tissues were rinsed with precooled saline and gently blotted dry with filter paper. The jejunum was fixed in 10 % formalin for further histopathological analysis. The liver, thymus, and spleen were weighed and stored at −80 °C. Immune organ indices were calculated using the following formula:

$$\mathrm{Organ index}=\mathrm{organ mass}\;\left(\mathrm{mg}\right)/\mathrm{animal body mass}\;\left(g\right).$$

### 2.4 Determination of serum inflammatory factors

Serum interleukin (IL)-1β and tumor necrosis factor (TNF)-α levels were measured using ELISA kits (Shanghai Xinle Biotechnology Co., Ltd., Shanghai, China) following the manufacturer's instructions.

### 2.5 Determination of the oxidative stress index

Hepatic alanine aminotransferase (ALT), catalase (CAT), superoxide dismutase (SOD) activities, and malondialdehyde (MDA) levels were determined using commercial kits (Nanjing Jiancheng Bioengineering Institute, Nanjing, China) following the manufacturer's instructions.

### 2.6 Histological characterization

Jejunal tissue was preserved in a 10 % tissue fixative, embedded in paraffin, and sectioned for histopathological analysis. Hematoxylin and eosin (HE) staining was conducted to assess tissue morphology, and the Alcian blue-periodic acid-Schiff (AB-PAS) method was employed to assess goblet cell distribution and mucin content. Images were captured using a light microscope at ×200 magnification (Nikon Eclipse Ci-L, Nikon, Tokyo, Japan).

### 2.7 Quantitative real-time PCR (qRT-PCR) analysis

Total RNA was extracted from spleen tissue using the MiniBEST Universal RNA Extraction Kit (TaKaRa, Dalian, China) and reverse-transcribed into cDNA using the PrimeScript RT Master Mix (TaKaRa) on a 9600 GeneAmp thermal cycler. qRT-PCR was conducted on a LightCycler 480 system (Roche, Basel, Switzerland) to quantify target gene expression. Relative expression levels were calculated using the 2^−ΔΔCt^ method. Primer sequences are listed in **Table S1**.

### 2.8 Quantification of SCFAs

Fecal samples (100 mg) were mixed with dilute sulfuric acid (1 μL) and diethyl ether (1 mL), centrifuged (10,000 ×*g*, 10 min, 4 °C). The supernatant was mixed with anhydrous sodium sulfate (250 mg) and centrifuged again under the same conditions. The supernatant was filtered through a 0.22 μm filter membrane and analyzed for SCFAs by gas chromatography (GC), as described previously (An et al., 2024). Briefly, GC analyses were performed on a GC-2030 system (Shimadzu, Kyoto, Japan) equipped with a flame ionization detector and an HP-INNOWAX capillary column (30 m × 0.25 mm × 0.25 μm; Agilent Technologies, Santa Clara, CA, USA). The oven temperature was initially set at 100 °C, ramped to 180 °C at 5 °C/min, and held for 4 min. Nitrogen, hydrogen, and air were used as the carrier and makeup gases at flow rates of 3, 4, and 3 mL/min, respectively.

### 2.9 Gut microbiota analysis

16S rRNA sequencing was performed according to a previously reported method (Liu et al., 2022). Genomic DNA was extracted using the CTAB or SDS method, and its purity and concentration were assessed by agarose gel electrophoresis. The diluted genomic DNA (1 ng/μL) was used as the template for PCR amplification. Operational taxonomic unit (OTU) analysis was performed to assess species richness, evenness, and the presence of shared or unique OTUs across samples or groups. Principal component analysis (PCA) was applied to evaluate the differences in community structure among samples or groups. A *t*-test analysis was used to identify significant differences in species composition between the groups.

### 2.10 Extraction and detection of serum metabolites

Serum metabolomics was employed to investigate the effects of LBP3 on serum metabolic profiles in immunosuppressed mice. Serum samples (50 μL) were mixed with extraction solvent (200 μL, methanol: acetonitrile = 1:1, *v*/v), sonicated (30 min), and centrifuged (10,000 ×*g*, 15 min, 4 °C). The supernatant was analyzed by LC-MS/MS according to previously established methods (Yang et al., 2024). For quality assurance and system stability, a pooled quality control (QC) sample was generated by mixing equal volumes of all individual samples. Target compounds were separated on an ACQUITY BEH C18 column (2.1 mm × 100 mm, 1.7 μm; Waters, Milford, MA, USA) using a Vanquish UHPLC system. The mobile phase consisted of solvent A (0.1 % formic acid in water) and solvent B (0.1 % formic acid in acetonitrile). The injection volume was 2 μL, and the column temperature was maintained at 40 °C.

### 2.11 Statistical analysis

Data were analyzed using SPSS 20.0 software. Results are presented as means ± SEM. Significant differences (*p* < 0.05) were evaluated using analysis of variance (ANOVA), followed by Duncan's multiple-range test. Biological and technical replicates were designed as follows: (a) jejunal tissues from three mice per group were sectioned and prepared on slides in triplicate, with the most representative section chosen for analysis; and (b) liver antioxidant activity and splenic cytokine levels were assessed in five mice per group, with each sample analyzed in triplicate.

## 3 Results

### 3.1 Effects of various LBP components on immune function in CTX-treated mice

**Fig. 1:**
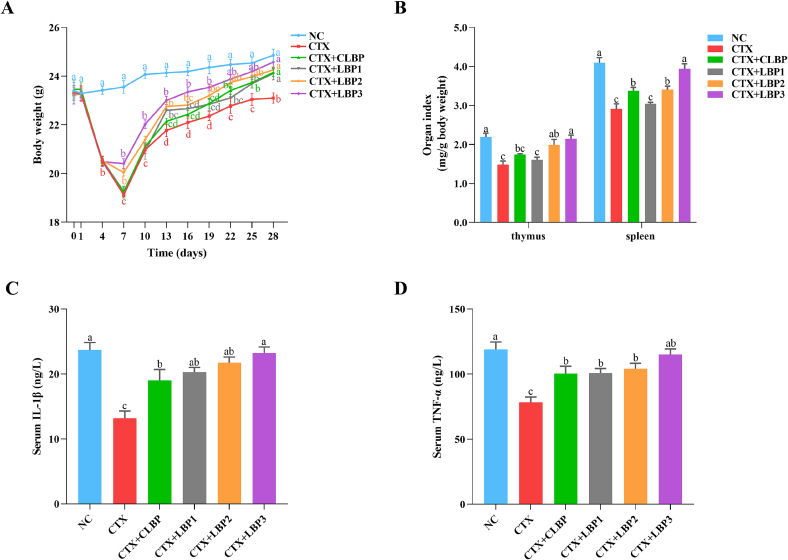
Effects of various LBP components on immune function in CTX-treated mice. (A) body weight, (B) organ index, (C) serum IL-1β levels, and (E) serum TNF-α levels. The different lowercase letters indicate a significant difference (*p* < 0.05) among different groups. The data are presented as the mean ± SEM (*n* ≥ 5).

Following three days of CTX administration, mice in all CTX-treated groups exhibited significant weight loss (approximately 12 %), confirming successful establishment of the immunosuppression model **(**Fig. 1A**)**. From days 4 to 7, the rate of weight loss in the CTX-treated mice decreased, although disparities were observed among the various intervention groups. Thereafter, all CTX-treated mice gradually regained weight, with LBPs-treated groups recovering significantly faster than the CTX group. By day 28, body weight in the CTX group remained below than that of the NC group, whereas LBPs-treated mice reached comparable levels. The trend in food intake was similar to that of body weight **(Fig. S1)**. Additionally, CTX markedly reduced thymus and spleen indices compared with NC mice (thymus: 1.5 ± 0.2 vs. 2.2 ± 0.2 mg/g body weight; spleen: 2.9 ± 0.3 vs. 4.1 ± 0.3 mg/g body weight) **(**Fig. 1B**)**, suggesting that CTX caused serious damage to both these organs. However, LBP2 (2.0 ± 0.3 mg/g body weight) and LBP3 (2.1 ± 0.2 mg/g body weight) interventions significantly enhanced the thymus index in CTX-treated mice. In comparison with the CTX group, the spleen indices for the CTX + CLBP (3.4 ± 0.2 mg/g body weight), CTX + LBP2 (3.4 ± 0.2 mg/g body weight), and CTX + LBP3 (3.9 ± 0.3 mg/g body weight) groups were significantly higher. In particular, the thymus and spleen indices for the CTX + LBP3 group recovered to those of the NC group, indicating that LBP3 was more effective in alleviating CTX-induced damage to immune organs.

Cytokines are soluble proteins produced by activated immune and certain non-immune cells that mediate intercellular communication and play critical roles in coordinating physiological processes (Schoenaker et al., 2018). As shown in Fig. 1C**&D**, serum IL-1β (13.2 ± 2.5 ng/L) and TNF-α (78.4 ± 9.2 ng/L) levels were notably lower in the CTX group than in the NC group (23.7 ± 2.6 ng/L, and 118.9 ± 13.0 ng/L, respectively), suggesting that CTX greatly suppressed the immune activity. However, LBPs intervention increased both cytokines to varying extents. Notably, only the CTX + LBP3 group restored IL-1β (23.2 ± 2.1 ng/L) and TNF-α (115.0 ± 9.6 ng/L) to levels comparable to those of the NC group, indicating that LBP3 exerts the most potent immunostimulatory effect among the other polysaccharide components.

### 3.2 Effects of various LBP components on oxidative stress and inflammatory response in CTX-treated mice

**Fig. 2:**
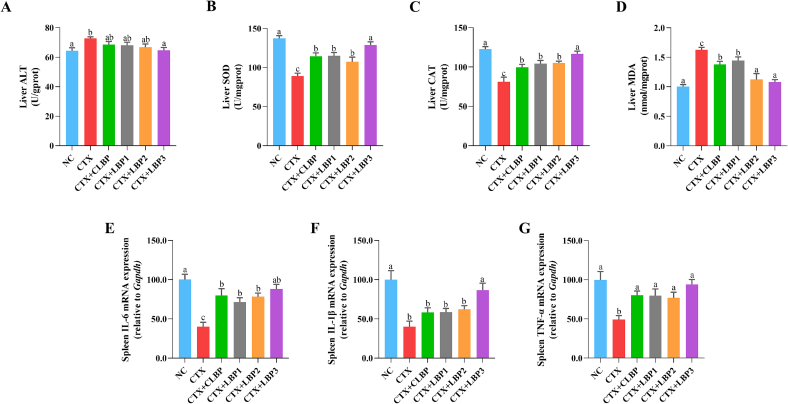
Effects of various LBP components administration on oxidative stress and inflammatory response in CTX-treated mice. (A) liver ALT activity, (B) liver SOD activity, (C) liver CAT activity, (D) liver MDA level, (E) spleen IL-6 expression, (F) spleen IL-1β expression, and (G) spleen TNF-α expression. The different lowercase letters indicate a significant difference (*p* < 0.05) among different groups. The data are presented as the mean ± SEM (n ≥ 5).

CTX treatment induces hepatotoxicity, with increased ALT activity typically closely linked to liver cell damage (Yin et al., 2022; Zhao et al., 2020). In this study, only LBP3 significantly reduced the CTX-induced elevation in ALT activity **(**Fig. 2A**)**. CTX-induced hepatotoxicity is attributed to cytotoxic metabolism, particularly acrolein, which promotes the generation of reactive oxygen species (ROS), resulting in oxidative stress and immune suppression (Ding, Yan, Peng, et al., 2019). SOD and CAT are essential antioxidant enzymes that protect cells from ROS-mediated damage. MDA is a byproduct of endogenous lipid peroxidation, and its accumulation can lead to DNA rearrangement and cell apoptosis (Ou et al., 2019). Relative to the NC group, the CTX group showed markedly elevated MDA levels and reduced SOD and CAT activities **(**Fig. 2B-D**)**, indicating that CTX impaired hepatic oxidative stress defenses. Compared to the CTX group, various LBP components significantly increased CAT and SOD activities and reduced MDA levels, especially in the CTX + LBP3 group, which recovered to the levels in the NC group. These results suggest that LBP3 possesses the greatest potential for ameliorating CTX-induced liver dysfunction in mice.

The spleen plays a critical role in immune surveillance and response by regulating cytokine secretion and overall immune function (Nie et al., 2023). To investigate the effects of various LBP components on spleen-related gene expression, qRT-PCR was performed **(**Fig. 2E-G**)**. Relative to the NC group, the expression levels of IL-6, IL-1β, and TNF-α were markedly reduced in the CTX group. However, treatment with various LBP components significantly upregulated IL-6 and TNF-α expression. Notably, only the CTX + LBP3 group restored IL-1β expression to a significant extent.

### 3.3 Effects of various LBP components on the jejunum histology and SCFAs production in CTX-treated mice

**Fig. 3:**
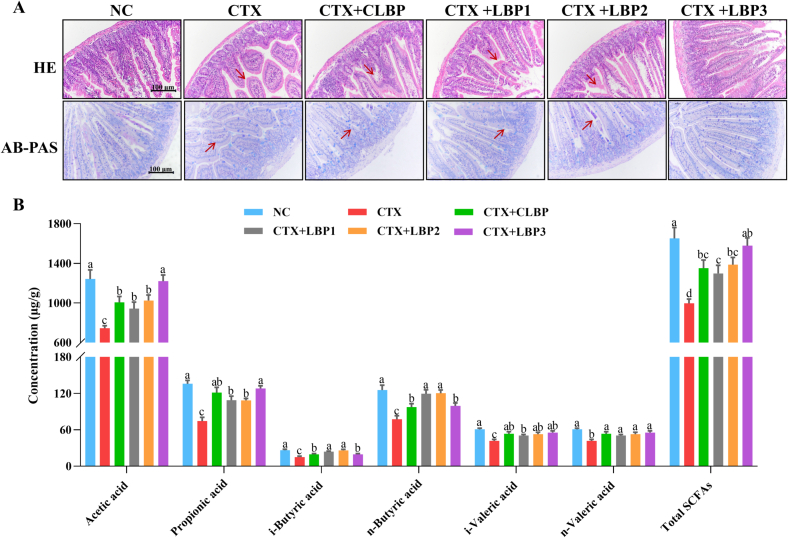
Effects of various LBP components on the jejunum histological changes and SCFAs production in CTX-treated mice. (A) morphological changes in the jejunum tissue (×200), and (B) SCFAs concentrations. The different lowercase letters indicate a significant difference (*p* < 0.05) among different groups. The data are presented as the mean ± SEM (n ≥ 5).

The intestinal epithelium serves as a vital physical barrier that protects against pathogen invasion while fostering essential interactions between the gut microbiota and immune system (Martini et al., 2017). To evaluate the effects of various LBP components on CTX-induced intestinal pathological damage, we performed H&E and AB-PAS staining. The jejunal tissue structure in the NC group appeared normal, with orderly villi, distinct boundaries, abundant goblet cells, and extensive mucus coverage **(**Fig. 3A**)**. In contrast, CTX-treated mice showed severe jejunal lesions, including villus atrophy, structural disintegration, and marked reductions in goblet cell numbers and mucus coverage. LBPs intervention resulted in varying degrees of restoration of intestinal mucosal damage, with longer and thicker villi, a more orderly arrangement, and increased goblet cell numbers and mucus coverage. These findings suggest that LBPs may protect the intestinal mucosa by promoting epithelial repair and enhancing goblet cell function.

Gut microbiota metabolizes polysaccharides into SCFAs, primarily acetic, propionic, and butyric acids, which are essential for immune regulation by modulating the intestinal immune environment, enhancing gut barrier function, and inhibiting inflammatory responses. As shown in Fig. 3B, CTX treatment markedly reduced the concentrations of individual SCFAs in mice feces, leading to the lowest total SCFAs concentration (995.9 ± 97.9 μg/L) in contrast to the NC group (1651.4 ± 248.3 μg/L). In contrast, various LBP components intervention significantly enhanced the total SCFAs concentration (1351.4 ± 182.4 μg/L for CLBP, 1298.7 ± 184.7 μg/L for LBP1, 1387.2 ± 160.6 μg/L for LBP2, and 1577.9 ± 178.2 μg/L for LBP3). However, their impact on the production of individual SCFAs varied. Specifically, LBP1 and LBP2 markedly enhanced i-butyric and n-butyric acids concentrations, with LBP2 showing the most pronounced increases (1.7- and 1.6-fold higher than the CTX group, respectively). In contrast, the CTX + LBP3 group exhibited significantly higher concentrations of acetic and propionic acids than the other LBPs intervention groups, with 1.6- and 1.7- times higher concentrations, respectively, than those in the CTX group.

### 3.4 Effects of various LBP components on gut microbiota composition

**Fig. 4:**
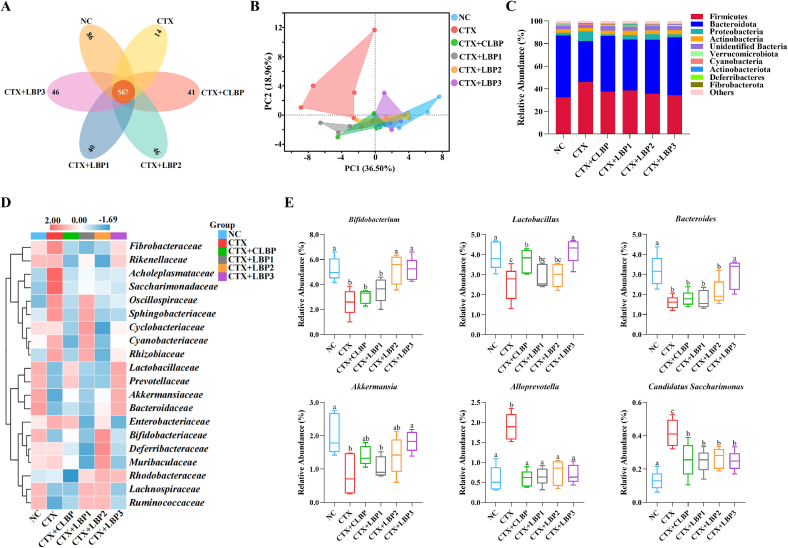
Effects of various LBP components on the gut microbiota in CTX-treated mice. (A) venn diagram, (B) PCA, (C) percentage of species at the phylum level, (D) heat map of bacterial community relative abundances at the family level, and (E) species level differential abundance plot. The different lowercase letters indicate a significant difference (*p* < 0.05) among different groups. The data are presented as the mean ± SEM (*n* = 5).

To explore the regulatory effects of various LBP components on gut microbiota in immunosuppressed mice, 16S rRNA gene sequencing was performed. Venn diagrams (Fig. 4A) illustrate shared and unique OTUs among the groups. The unique OTU counts in the NC, CTX, CTX + CLBP, CTX + LBP1, CTX + LBP2, and CTX + LBP3 groups were 86, 14, 41, 40, 46, and 46, respectively. CTX treatment markedly reduced unique OTUs, whereas various LBP components exhibited varying degrees of recovery in unique OTU counts. α-Diversity of gut microbiota was quantified via the Chao, ACE, Shannon, and Simpson indices. Various LBP interventions significantly increased the Chao and ACE indices **(Fig. S2)**. To explore the differences in the gut microbiota structure between the groups, PCA was performed **(**Fig. 4B**)**. The results showed a distinct separation between the NC and CTX groups, whereas the various LBP intervention groups were situated closer to the NC group, indicating that LBPs intervention effectively restored the CTX-induced microbiota imbalance.

To obtain a deeper insight into the specific changes in the microbiota, the taxa composition of the gut microbiota was analyzed across the different groups. At the phylum level, gut microbiota predominantly consisted of Bacteroidetes, Firmicutes, and Proteobacteria **(**Fig. 4C**)**. CTX markedly increased the relative abundance of Firmicutes and Proteobacteria, while decreasing Bacteroidetes and Verrucomicrobiota, resulting in Firmicutes becoming the dominant phylum. Various LBP interventions reversed these changes, significantly reducing the relative abundance of Firmicutes and Proteobacteria, while increasing the relative abundance of Bacteroidetes. Additionally, CLBP and LBP3 interventions notably elevated the relative abundance of Verrucomicrobiota. At the family level, compared with the NC group, the CTX group greatly enhanced the relative abundance of *Saccharimonadaceae* and *Acholeplasmataceae*, whereas the relative abundance of *Lactobacillaceae*, *Lachnospiraceae*, *Prevotellaceae*, *Bacteroidaceae*, *Ruminococcaceae*, and *Akkermansiaceae* was notably reduced **(**Fig. 4D**)**. In comparison with the CTX group, intervention with various LBP components exhibited differential effects: CTX + CLBP group markedly improved the relative abundance of *Lactobacillaceae* and *Prevotellaceae*; CTX + LBP1 and CTX + LBP2 groups notably enhanced the relative abundance of *Lachnospiraceae* and *Ruminococcaceae*; and CTX + LBP3 group significantly increased the relative abundance of *Lactobacillaceae*, *Prevotellaceae*, *Bacteroidaceae*, and *Akkermansiaceae*. In addition, all LBP treatments dramatically decreased the relative abundance of *Saccharimonadaceae* and *Acholeplasmataceae*.

In addition, notable changes at the genus level were determined using intergroup *t*-test analysis **(**Fig. 4E**)**. Compared with the CTX group, various LBP components notably reduced the relative abundance of *Alloprevotella* and *Candidatus Saccharimonas*, while differentially increasing the relative abundance of *Bifidobacterium*, *Lactobacillus*, *Bacteroides*, and *Akkermansia*.

### 3.5 Regulation of serum metabolite profiles in CTX-treated mice by LBP3

**Fig. 5:**
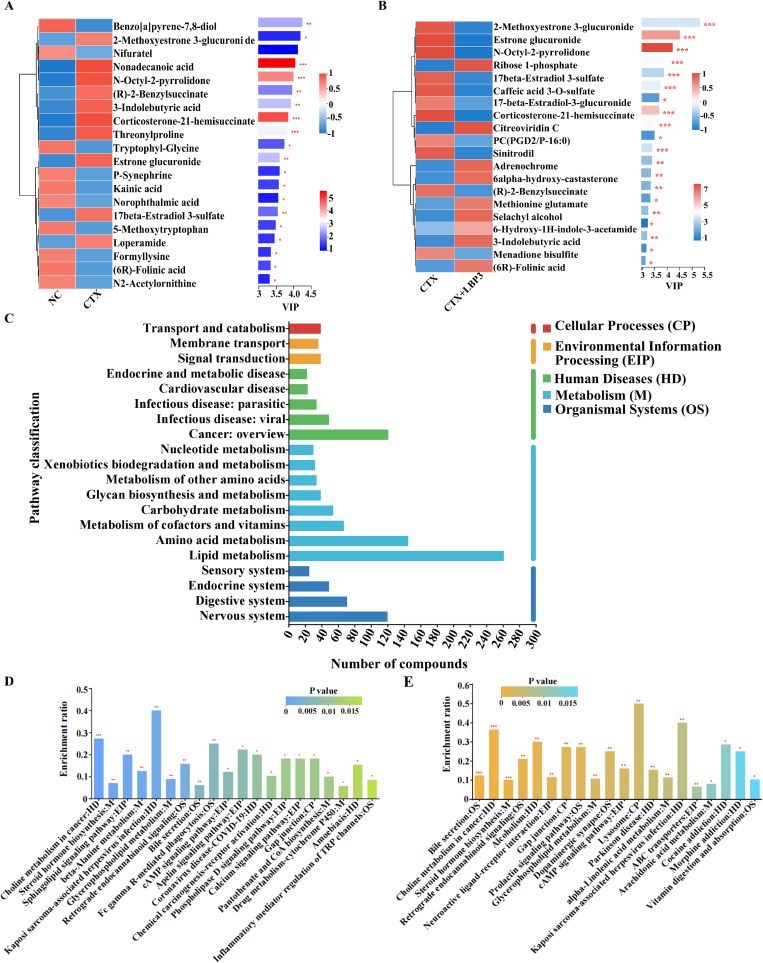
Regulation of serum metabolite profiles in CTX-treated mice by LBP3. Heatmap and VIP scores of metabolites comparing the (A) NC and CTX groups and (B) CTX and CTX + LBP3 groups; (C) KEGG pathway classification analysis; Enriched metabolic pathways of differential compounds between the (D) NC and CTX groups and (E) CTX and CTX + LBP3 groups.

Given the significant potential of the L. *barbarum* arabinogalactan component LBP3 in enhancing immune function and regulating the homeostasis of gut microbiota, untargeted metabolomic analysis was performed to explore the changes in the serum metabolites in immunosuppressed mice after LBP3 administration. Partial least squares discriminant analysis (PLS-DA) was applied to distinguish metabolic differences among the NC, CTX, and CTX + LBP3 groups. A clean separation was observed in the PLS-DA score plots for both the positive and negative ion modes **(Fig. S3 A&B)**, indicating that LBP3 intervention significantly altered serum metabolite profiles compared with the NC and CTX groups. To further identify the key metabolites affected by LBP3, the metabolites with the top 20 VIP scores (VIP > 1.0) based on the PLS-DA model were selected as potential biomarkers and analyzed using a heatmap and VIP score plots. As shown in Fig. 5A, 20 metabolites exhibited significant differences between the NC and CTX groups, with 10 downregulated and 10 upregulated in the CTX group. Among them, four metabolites with higher VIP scores (VIP ≥ 4.0), including benzo[*a*]pyrene-7,8-diol (down-regulated, VIP = 4.2), 2-methoxyestrone 3-glucuronide (up-regulated, VIP = 4.2), nifuratel (down-regulated, VIP = 4.1), and nonadecanoic acid (up-regulated, VIP = 4.0), were considered the most important biomarkers in the CTX group. However, only 19 metabolites exhibited notable differences between the CTX and CTX + LBP3 groups **(**Fig. 5B**)**. Compared with the CTX group, 8 metabolites were enriched and 11 were decreased in the CTX + LBP3 group. Notably, four metabolites with VIP ≥ 4.0 included 2-methoxyestrone 3-glucuronide (down-regulated, VIP = 5.3), estrone glucuronide (down-regulated, VIP = 4.5), N-octyl-2-pyrrolidone (down-regulated, VIP = 4.2), and ribose 1-phosphate (up-regulated, VIP = 4.0), were identified as the most important biomarkers associated with LBP3 intervention.

Next, KEGG metabolic pathway analysis was performed on the identified metabolites. The metabolites were primarily mapped to five KEGG primary pathways **(**Fig. 5C**)**: cellular processes (CP), environmental information processing (EIP), human diseases (HD), metabolism (M), and organismal systems (OS). Most of the metabolites were categorized under M, with the largest group being lipid metabolism (*n* = 261), followed by amino acid metabolism (*n* = 145) and the metabolism of cofactors and vitamins (*n* = 67). For the terms “OS”, “HD”, “EIP”, and “CP”, the most prominent categories were nervous system (*n* = 120), cancers: overview (*n* = 121), signal transduction (*n* = 39), and transport and catabolism (n = 39), respectively. Further metabolic pathway enrichment analysis revealed that, compared with the NC group, the CTX group showed enrichment in 20 specific pathways. Similarly, after LBP3 intervention, the CTX + LBP3 group was also significantly enriched in 20 pathways relative to the CTX group **(**Fig. 5D**&E)**. Notably, eight metabolic pathways were shared between the NC-CTX and CTX-CTX + LBP3 groups, including choline metabolism in cancer, steroid hormone biosynthesis, kaposi sarcoma-associated herpesvirus infection, glycerophospholipid metabolism, retrograde endocannabinoid signaling, bile secretion, cAMP signaling pathway, and gap junctions. Finally, a heatmap analysis was performed on the differentially expressed metabolites (*p* < 0.01, Top 30) in the NC, CTX, and CTX + LBP3 groups **(Fig. S3C)**. In contrast to the NC group, CTX notably reduced the levels of metabolites, such as inosine, pentadecanoic acid, propionylcarnitine, and fucoxanthin, whereas LBP3 intervention significantly reversed these changes. These results indicate that LBP3 intervention can effectively improve metabolic disorders in immunosuppressed mice.

### 3.6 Correlation between the differential gut microbiota and host parameters

**Fig. 6:**
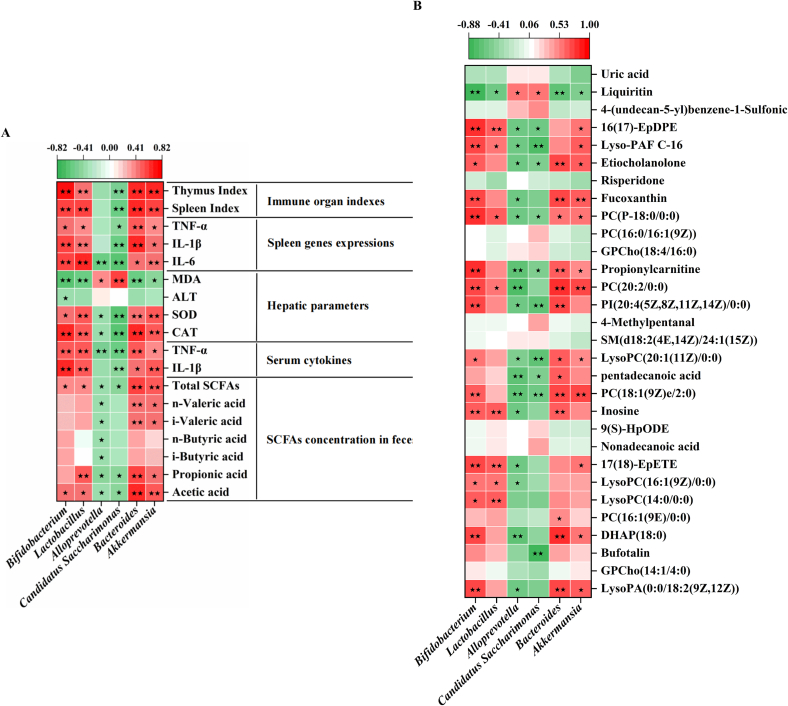
Correlation analysis between characteristic bacteria and (A) immune traits, (B) serum metabolites in CTX-treated mice. Colors of squares represent the R-value of Spearman's correlation. ★ and ★★ indicate the significance of association at levels of *p* < 0.05 and *p* < 0.01, respectively.

To further explore the associations between the notably altered microbial communities at the genus level and immune response parameters, along with serum differential metabolites, Spearman's correlation analysis was conducted. The results demonstrated that the relative abundance of significantly altered bacteria closely correlated with immune organ indices, relative gene expression levels in the spleen, liver parameters, and SCFAs concentrations. Notably, *Bifidobacterium*, *Lactobacillus*, *Bacteroides*, and *Akkermansia*, which were significantly enriched following LBP interventions, showed strong positive correlations with immune traits. In contrast, *Alloprevotella* and *Candidatus Saccharimona*s, which were significantly reduced, exhibited strong negative correlations with immune traits **(**Fig. 6A**)**. Furthermore, the gut microbiota influences serum metabolite levels by regulating intestinal metabolites, showing significant correlations **(**Fig. 6B**)**.

## 4 Discussion

Impaired immune function is closely associated with the development of various chronic diseases, such as infections, cancer, and metabolic disorders (Andreou & Papaneophytou, 2025; Vuscan et al., 2024). In the post-COVID era, the immune-boosting health industry is expected to enter an “era of universal need.” Dietary supplementation with natural bioactive compounds, particularly polysaccharides, has emerged as an effective, accessible, and sustainable strategy for modulating immune function (Wang et al., 2025; Zhao et al., 2025). Previous studies have shown that even subtle structural differences in polysaccharides can significantly influence their immunoregulatory effects. Chen, Wang, et al. (2024) found that fermented *Gastrodia Ganoderma* polysaccharides (FRP), primarily composed of GlaA (65.7 %), exhibited superior free radical scavenging (ABTS, DPPH, hydroxyl radical scavenging, and ferric reducing antioxidant power) compared with non-fermented polysaccharides (RP), which mainly contain glucose (Glc, 66.5 %). Additionally, FRP more effectively inhibited LPS-induced ROS production in anti-inflammatory cell models and demonstrated higher affinity for inflammatory markers, thereby reducing immune damage. These enhanced immunoprotected effects were attributed to the unique configuration of the carboxyl and uronic acid residues in the FRP. Jiang et al. (2021) observed that the degraded, lower-Mw fucoidan from the sea cucumber *Stichopus chloronotus* (113.1 kDa, Fuc—Sc) exhibited stronger immunostimulatory activity than the high-Mw Fuc-Sc component (247.3 kDa) by promoting the production of TNF-α, IL-6, and IL-10. This effect likely results from the fact that polysaccharides with lower Mw can more effectively bind to TLR2 and TLR4 on macrophage surfaces, activating NF-ҡB signaling pathway and promoting the release of inflammatory and immunomodulatory cytokines. Despite these advances, the effective isolation and purification of polysaccharides remains challenging due to their structural complexity, and the structure–function relationships underlying immune modulation require further investigation.

LBPs, as plant-derived polysaccharides, have been extensively investigated for their diverse functional activities. Gong et al. (2018) purified LBP1, LBP2, and LBP3 on a Sephadex G-100 column to obtain LBGP1, LBGP2, and LBGP3, and demonstrated that these fractions exhibited distinct antioxidant and immunomodulatory activities. For superoxide radical scavenging, the EC₅₀ values of LBP, LBGP1, LBGP2, and LBGP3 were 1.3, 2.0, 1.3, and 2.1 mg/mL, respectively, with scavenging activities of 68.7 %, 65.1 %, 68.7 %, and 60.1 % at 3.0 mg/mL. For hydroxyl radical scavenging, the EC₅₀ values were 1.0, 1.9, 0.9, and 2.1 mg/mL, while for DPPH radical scavenging the rates reached 92.3 %, 74.7 %, and 85.5 % at 2.0 mg/mL. In addition, LBGP3 showed the strongest immunomodulatory effect, likely due to its arabinogalactan structure, which has been reported to activate TLR4 receptors on macrophages. For example, all four fractions enhanced the phagocytic activity of RAW 264.7 cells in a dose-dependent manner (12.5–200 μg/mL), with LBGP3 achieving a phagocytosis index of 1.9 at 200 μg/mL, comparable to the positive control (LPS). Similarly, nitric oxide production was stimulated in a concentration-dependent manner, with LBP, LBGP1, LBGP2, and LBGP3 inducing levels of 11.6, 16.3, 20.0, and 22.9 μmol/L, respectively, at 200 μg/mL. In the present study, we found that, compared with other LBP components, the LBP3 component with arabinogalactan structure most effectively mitigated CTX-induced weight loss, protected immune organs (improving the immune organ index and repairing the jejunal damage), stimulated the secretion of immune-related cytokines (IL-6, IL-1β, and TNF-α), and prevented hepatotoxicity (CAT, SOD, ALT, and MDA). These findings are consistent with previous reports demonstrating the significant immunomodulatory effects of arabinogalactans (Dai et al., 2024; Gu et al., 2024).

As non-digestible polysaccharides, LBPs are metabolized by the gut microbiota to generate SCFAs, which represent a key mechanism underlying their immunoregulatory effects. Acetate promotes IL-6 production by activating GPR43, which in turn stimulates sIgA synthesis and secretion, strengthening gut mucosal immunity (Wu et al., 2017). Propionate supports the growth and differentiation of T lymphocytes and increases IL-10 expression. Butyrate regulates inflammation by activating GPR109A and serving as an energy source for intestinal epithelial cells, thereby influencing the proliferation and metabolism activity of both intestinal epithelial and immune cells (Priyadarshini et al., 2018). Studies have shown that different structural characteristics of polysaccharides have a considerable impact on the production of specific SCFAs. Chen et al. (2022) found that *Fortunella margarita* polysaccharides yielded higher acetate and propionate levels during in vitro fermentation compared with Glc and inulin. This may be attributed to their composition, which includes Gal, Ara, rhamnose (Rha), and GlaA. Mortensen et al. (1988) reported that Rha, Ara, xylose (Xyl), and GlaA significantly increased the propionate concentration, whereas sorbitol, GlaA, and glucuronic acid (GlcA) were more effective in increasing the butyrate concentration. Some studies have also reported that low-Mw polysaccharides may facilitate SCFAs production due to their loose structures and greater exposure of active groups. Chen et al. (2019) found that low-Mw *Chimonobambusa quadrangularis* polysaccharides more effectively elevated the concentrations of acetate, propionate, and butyrate, thereby benefiting the intestinal ecosystem and probiotic activity. However, some researchers argued that Mw alone does not necessarily correlate with SCFA production. Xing et al. (2021) found that high-Mw (2265.0 kDa) *Dendrobium officinale* polysaccharides were more effective than low-Mw (1255.6 kDa) in increasing the concentrations of acetate and propionate. Overall, these studies indicate that the structures of polysaccharides determine their specific gut metabolic pathways. Acetate is primarily produced through the fermentation of Gal and GlaA, propionate mainly from Glc and Ara, and butyrate predominantly from GlaA (Chen et al., 2016). In this study, various LBP components effectively alleviated the decrease in total SCFAs concentrations after treatment with CTX. However, their effects on the production of individual SCFAs varied. Compared with the other polysaccharide groups, the CTX + LBP3 group exhibited higher concentrations of acetic and propionic acids, whereas the butyric acid concentration was notably higher in the CTX + LBP1 and CTX + LBP2 groups. These differences could be attributed to LBP3 having a uniform arabinogalactan structure, enriched in Ara and Gal, whereas LBP1 and LBP2 contain higher GlaA content. Previous studies have also reported that LBP3 selectively elevates acetic and propionic acids concentrations, whereas pectin polysaccharides rich in galacturonic acid (GalA) structures preferentially increase butyric acid concentration in mice feces (Zhao et al., 2023). Together, these findings support the notion that polysaccharide structural variation is a major determinant of SCFAs production (An et al., 2024).

Polysaccharides with different structural features can be selectively utilized by specific gut microbiota through the action of carbohydrate-active enzymes (CAZymes). Studies on longan and oat polysaccharides have revealed that longan polysaccharides strongly promote the growth of *Enterococcus faecium*, *Lactobacillus casei*, and *Lactobacillus acidophilus*, whereas oat polysaccharides exert a comparatively weaker effect. This difference may be attributed to the presence of Glc, mannose (Man), and Ara in longan polysaccharides, compared with the predominantly Glc composition of oat polysaccharides (Wang, Zhang, et al., 2020). Li et al. (2023) found that four galactans with distinct structural features (porphyran, agarose, carrageenan, and arabinogalactan) could be utilized by *Bacteroides*. Differently, porphyran can be metabolized by *Lactobacillus* and *Bifidobacterium*; carrageenan promotes the growth of *Prevotella*, *Megamonas*, and *Bifidobacterium*; whereas arabinogalactan enhances the proliferation of *Lactobacillus* and *Roseburia.* Wang, Shao, et al. (2020) isolated three types of homogeneous polysaccharides (SY01–21, SY01–22, and SY01–23) containing Rha, Gal, and Ara from mulberry leaves and found that SY01–23 had a significantly stronger effect in increasing the relative abundance of *Bacteroides cellulosilyticus* and *Bacteroides ovatus* than the other two polysaccharides, which may be due to its higher content of GalA and GlcA. Additionally, Li et al. (2022) reported that low-Mw blackberry polysaccharides (177.4 kDa) stimulated *Bacteroidetes* proliferation more effectively than medium-Mw fractions (363.9 kDa). Conversely, medium-Mw konjac glucomannan (KGM, 757.1 kDa) is more effective than low-Mw (87.3 kDa) KGM in enriching gut microbiota diversity and significantly increasing the relative abundance of *Ruminococcus* and *Lachnoclostridium* (Deng et al., 2020). These findings highlight the complexity of gut microbiota in polysaccharide metabolism, demonstrating that different microorganisms possess specific enzymes and regulatory mechanisms that enable selective degradation of various polysaccharides. In this study, various LBP components also showed distinct effects in alleviating the gut microbiota imbalance in CTX-treated immunosuppressed mice. LBP1 and LBP2 interventions markedly increased the relative abundance of *Lachnospiraceae* and *Ruminococcaceae*, whereas no significant changes were observed in the CTX + CLBP and CTX + LBP3 groups. Moreover, LBP3 intervention significantly enhanced the proliferation of *Lactobacillaceae*, *Prevotellaceae*, *Bacteroidaceae*, and *Akkermansiaceae*, surpassing the effects of the other LBP groups. *Lactobacillaceae* and *Bacteroidaceae* encode a variety of CAZymes that selectively degrade and metabolize polysaccharides into acetic acid, while *Prevotellaceae* plays a key role in dietary fiber hydrolysis, converting polysaccharides into propionic acid via the succinate pathway (Louis & Flint, 2017). These activities likely contribute to the elevated acetic and propionic acids concentrations observed in the CTX + LBP3 group. Studies have revealed that *Prevotellaceae* is strongly correlated with the cytokine TGFB3, which regulates the gut barrier function (B et al., 2018). *Lachnospiraceae* is considered protective against inflammatory bowel disease. Decreased abundance of *Ruminococcaceae* may lead to an imbalance between Treg and Th17 cells (Han et al., 2018). Furthermore, LBP3 intervention notably enhanced the proliferation of *Bifidobacterium*, *Lactobacillus*, *Bacteroides*, and *Akkermansia* to the greatest extent. *Bifidobacterium* contain L-ribulose-5-phosphate 4-epimerase (AraD), which is a key factor in the regulation of Ara metabolism. It can effectively utilize Ara to promote growth (Arzamasov et al., 2018), and its main product, acetic acid, helps inhibit intestinal pathogens (Riviere et al., 2016). *Bacteroides* enhance immune defense by significantly increasing TNF-α production and restoring the ability of dendritic cells to stimulate T-cell expansion (Gauffin Cano et al., 2012). *Lactobacillus* strengthen immune function through interactions with gut-associated immune cells, upregulating CD206 and TLR2 receptors in macrophages and dendritic cells (Galdeano & Perdigón, 2006). *Akkermansia* can bypass T-independent IgA and enhance T-dependent immune reactions by penetrating the mucus layer and adhering to the intestinal epithelium, potentially modulating immune cells in Peyer's patches (Derrien et al., 2011).

Host metabolism encompasses a range of metabolic processes controlled by the host genome, and metabolic activities driven by the microbial genome. Metabolites generated by gut microbiota can enter the circulatory system, influencing host metabolism and overall health (Liu et al., 2025). Crucial metabolic pathways contributing to immune metabolism include glycolysis, the citrate cycle, fatty acid oxidation and synthesis, amino acid metabolism, and pentose phosphate pathway (Sun et al., 2023). In this study, serum metabolomic analysis revealed significant differences among the NC, CTX, and CTX + LBP3 groups, with these differences primarily involving metabolic pathways related to lipid metabolism, amino acid metabolism, and cancers: overview. Additionally, LBP3 intervention notably reversed the CTX-induced decreases in inosine, pentadecanoic acid, propionylcarnitine, and fucoxanthin. Inosine, a purine metabolite produced by *Akkermansia muciniphila* and *Bifidobacterium pseudolongum*, activates immune cells and promotes metabolic processes (Lu et al., 2022). Guo et al. (2023) demonstrated that inosine treatment could inhibit the release of inflammatory mediators via modulation of the NF-ҡB pathway, while also boosting antioxidant enzyme activity by regulating the Nrf2 pathway. Pentadecanoic acid, a saturated fatty acid with an odd‑carbon chain, known for its metabolic regulatory properties, is a key downstream metabolite produced by *Parabacteroides distasonis* during inulin metabolism. It can effectively restore the gut barrier function and reduce the production of serum lipopolysaccharides and proinflammatory cytokines in the liver (Wei et al., 2023). Propionylcarnitine, which exhibits a strong positive correlation with the abundance of *Desulfovibrionaceae*, can scavenge superoxide anions, prevent linoleic acid lipid peroxidation, and protect pBR322 DNA from H_2_O_2_-induced cleavage under UV photolysis (Feng et al., 2024; Vanella et al., 2000). Fucoxanthin significantly increases the proportion of *Parabacteroides* and *Bacteroides*, thereby enhancing gut microbiota functions related to glycan biosynthesis and metabolism, and improving the digestive, endocrine, and immune systems (Guo et al., 2024). Collectively, these findings emphasize the complex interplay among microbial metabolites, host metabolism, and immune regulation. LBP3 appears to restore metabolic homeostasis and strengthen immune responses by targeting key metabolites and microbial pathways disrupted by CTX treatment. Future studies will focus on cecal and fecal metabolites to further elucidate the differential immunomodulatory effects of various LBP components in immunosuppressed mice.

In this study, technical replicates were not performed for serum cytokines, fecal SCFAs, gut microbiota, and serum metabolomics measurements. This decision was made because the analytical platforms and procedures employed are highly reproducible and widely validated, while biological variation represents the primary source of experimental differences. Specifically, the ELISA kits employed had high sensitivity (0.5 ng/L) and reproducibility (intra-assay variation <10 %, inter-assay variation <12 %). For metabolomics, high-resolution LC-MS/MS instruments provide highly consistent measurements under fixed operating conditions, and the use of pooled QC samples together with stringent control of batch effects further minimized potential technical variation and ensured analytical reliability (Di et al., 2024; Qi et al., 2025). Fecal SCFAs were analyzed on the Shimadzu GC-2030 system under fixed and standardized conditions (injection volume, column, and temperature program), which greatly limited technical bias. For gut microbiota profiling, current high-throughput sequencing workflows are highly standardized, and when sequencing depth is sufficient, technical replicates yield nearly identical results, whereas biological differences remain the main driver of community variation (He et al., 2025; Jia et al., 2024; Yu et al., 2024). At the same time, standardized procedures were strictly followed for DNA extraction, library preparation, sequencing, and bioinformatics analysis to further minimize technical bias. Future work will employ metagenomic approaches to determine the species-level effects of LBPs in immunosuppressed mice and validate these findings through fecal microbiota transplantation. Moreover, advancing the isolation and purification of LBPs, particularly the LBP3 component, with its arabinogalactan structure, remains essential. Efforts to isolate its primary side chains and clarify their specific roles in regulating the gut microbiota and promoting targeted SCFA production will be essential. Although various LBP components promote SCFAs generation by interacting with characteristic bacterial populations, the intermediate products, action sites, and underlying mechanisms of this process require further elucidation.

## 5 Conclusion

In this study, the immunomodulatory effects of various LBP components and their structure-function relationships were explored based on the steady-state regulation of gut microbiota. The results demonstrated that various LBP components contributed to restoring body weight, protecting immune organs, promoting the secretion of immune-related cytokines, and alleviating hepatic oxidative stress. Among these, the arabinogalactan component LBP3 showed the highest immunomodulatory activity. Various LBP components distinctively altered the gut microbiota composition and SCFAs production in immunosuppressed mice, which could be a crucial factor contributing to the differences in their immunoregulatory effects. Serum metabolomics further revealed that LBP3 intervention significantly increased metabolite levels, including inosine, pentadecanoic acid, propionylcarnitine, and fucoxanthin, which were positively associated with the notably altered microbial communities. These findings provide novel insights into the mechanism by which LBPs exert immunomodulatory effects through gut microbiota regulation.

## CRediT authorship contribution statement

**Xin An:** Writing – review & editing, Writing – original draft, Methodology, Investigation, Formal analysis. **Yuan Chen:** Software, Data curation. **Yufei Chu:** Software, Data curation. **Mengjia Zhang:** Validation, Methodology. **Ye Liu:** Validation, Methodology. **Yuejuan Zhao:** Investigation. **Shan Wu:** Validation, Funding acquisition, Conceptualization. **Qian Liu:** Visualization, Validation, Supervision, Funding acquisition, Conceptualization.

## Funding

This work was supported by the 10.13039/501100001809National Natural Science Foundation of China (No. 32001697); Shaanxi Province Innovation Ability Support Plan-Youth Science and Technology Rising Star Project (Talent) (No. 2024ZC-KJXX-071); Yulin Science and Technology Plan Project in Shaanxi Province (No. 2023-CXY-222).

## Declaration of competing interest

The authors declare that they have no known competing financial interests or personal relationships that could have appeared to influence the work reported in this paper.

## Data Availability

Data will be made available on request.

## Appendix A Supplementary data

Supplementary material Image 1
