# Supplementary material for: Differential regulatory effects of Lycium barbarum polysaccharide components in cyclophosphamide-treated immunosuppressed mice: reshaping of the gut microbiota
Source: Food Chem (Oxf). 2025 Oct 3;11:100305. doi: 10.1016/j.fochms.2025.100305 (PMC12546882; doi:10.1016/j.fochms.2025.100305)
Supplement: Supplementary file 1 — Supplementary material [file mmc1.docx]

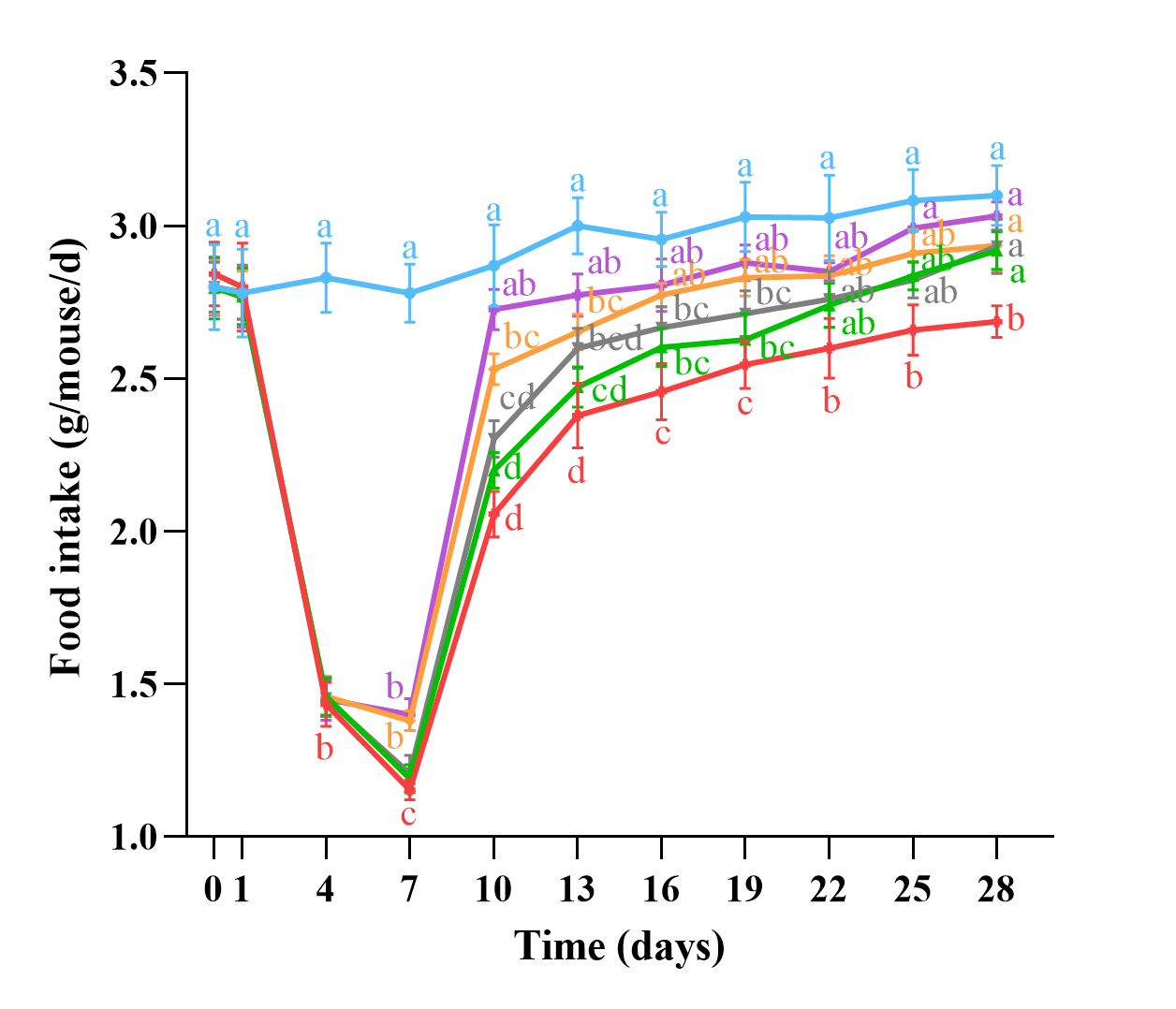


**Fig. S1** Effects of various LBP components on food intake in CTX-treated mice. The different lowercase letters indicate a significant difference (*p* <0.05) among different groups. The data are presented as the mean ± SEM (n ≥6).


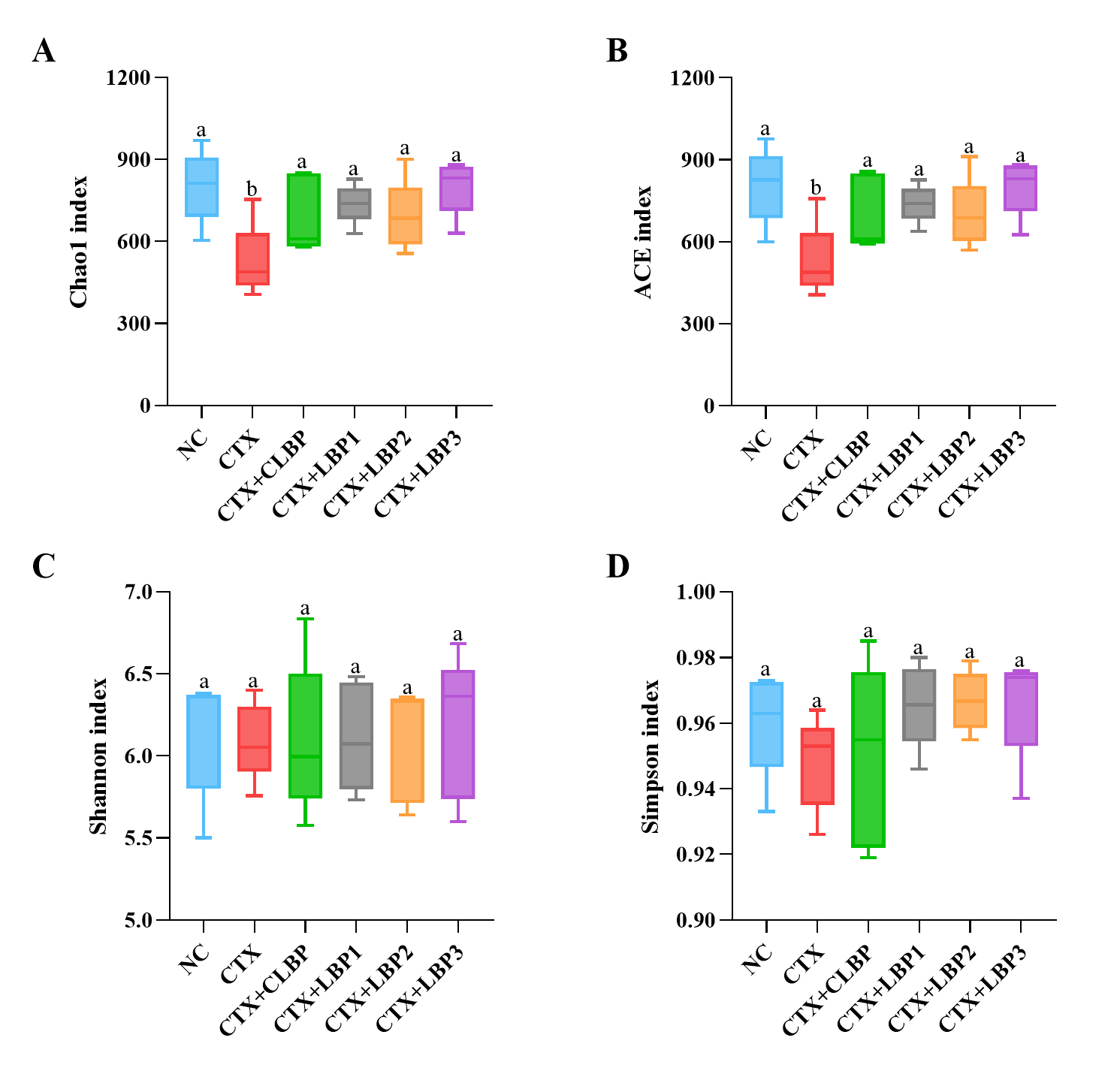


**Fig. S2** Effects of various LBP components administration on alpha diversity of gut microbiota in CTX-treated mice. (A) chao1 index, (B) ace index, (C) shannon index, and (D) simpson index. The different lowercase letters indicate a significant difference (*p* <0.05) among different groups. The data are presented as the mean ± SEM (n =5).


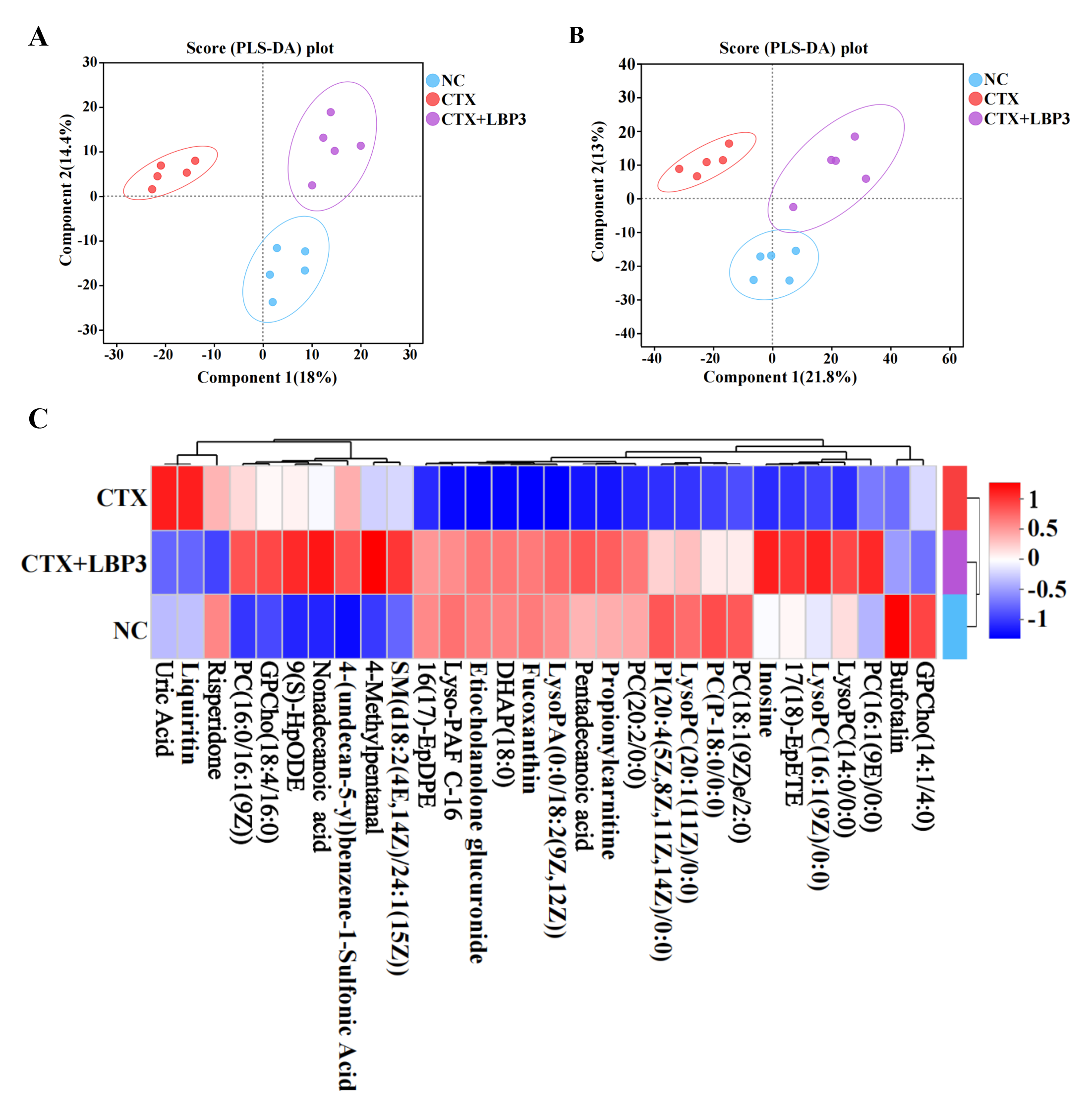


**Fig. S3** Effects of LBP3 on the serum metabolic profile in CTX-treated mice. PLS-DA plots of metabolites in the (A) positive and (B) negative ion models, and (C) thermogram of serum differential metabolites changes. The data are presented as the mean ± SEM (n =5).

**Table S1**

Target genes and primers sequence for qRT-PCR.

| Gene | Forward primer (5′-3′) | Reverse primer (5′-3′) |
| --- | --- | --- |
| *IL-1β* | AGCTTCAAATCTCGCAGCAG | TCTCCACAGCCACAATGAGT |
| *Tnf-α* | CCAGCCGATGGGTTGTACCT | TGACGGCAGAGAGGAGGTTG |
| *IL-6* | AGCCAGAGTCCTTCAGAGAG | GCCACTCCTTCTGTGACTCC |
| *Gapdh* | acagcaacagggtggtggac | tttgagggtgcagcgaactt |
